# Effects of resting modes on human lumbar spines with different levels of degenerated intervertebral discs: a finite element investigation

**DOI:** 10.1186/s12891-015-0686-z

**Published:** 2015-08-24

**Authors:** Ruoxun Fan, He Gong, Sen Qiu, Xianbin Zhang, Juan Fang, Dong Zhu

**Affiliations:** Department of Engineering Mechanics, Nanling Campus, Jilin University, Changchun, 130025 P. R. China; State Key Laboratory of Automotive Simulation and Control, Jilin University, Changchun, 130025 P. R. China; Department of Orthopedic Surgery, No. 1 Hospital of Jilin University, Changchun, 130025 People’s Republic of China

## Abstract

**Background:**

The negative effect of long-term working load on lumbar is widely known. However, insertion of different resting modes on long-term working load, and its effects on the lumbar spine is rarely studied. The purpose of this study was to investigate the biomechanical responses of lumbar spine with different levels of degenerated intervertebral discs under different working-resting modes.

**Methods:**

Four poroelastic finite element models of lumbar spinal segments L2-L3 with different grades of disc degeneration were developed. Four different loading conditions represented four different resting frequencies, namely, no rest, one-time long rest, three-time moderate rests, and five-time short rests, on the condition that the total resting time was the same except in the no rest mode. Loading amplitudes of diurnal activities included 100 N, 300 N, and 500 N.

**Results:**

With increasing resting frequency, the axial effective stress and fluid loss decreased, whereas the pore pressure and radial displacement increased. Under different resting frequencies, the changing rate of each biomechanical parameter was different.

**Conclusions:**

Under a situation of fixed total resting time, high resting frequency was advisable. If sufficient resting frequency was unavailable for healthy people as well as patients with mildly and moderately degenerated intervertebral discs, they could similarly benefit from relatively less resting frequencies. However, one-time rest will not be useful in cases where intervertebral discs were seriously degenerated. Reasonable working-resting modes for different degrees of disc degeneration, which could assist patients achieve a better restoration, were provided in this study.

## Background

Intervertebral discs are pads of hydrated fibrocartilage comprising gelatinous nucleus and fiber-reinforced annulus fibrosus. These discs lie between vertebral bodies, and ensure the flexibility of spine as well as the transmission of load by maintaining the balance between internal osmotic pressure and external pressures. In this process, fluid flow, which causes variations in disc height and proteoglycans content, is an important step [1–3]. However, intervertebral discs often suffer from degeneration because of aging and poor working environment, and it could be other plausible reasons for disc degeneration, such as genetic inheritance, inadequate metabolite transport, and loading history, which may directly change the normal hydrated environment and fluid flow and indirectly result in instability and reduction of the carrying capacity [1, 4–6]. Thus, investigating the changes of biomechanical parameters for different degrees of disc degeneration to explore relevant mechanism of disc degeneration and search for effective treatment strategies has significant value.

Disc degeneration is a progressive process that involves two changes: exterior geometry change that includes a decreased disc height, growth of osteophyte, and diffused border between annulus and nucleus [7–10] and interior material change that consists of increased nucleus stiffness, reduced water content, and changed permeability [11–13]. The biomechanical behavior of lumbar is greatly influenced by these changes. The results of compressive tests for normal and degenerated discs showed that the segmental motion increases with disc degeneration, but decreases when the degeneration advances to a severe degree [14–16]. An *in vivo* study for volunteers with normal and degenerated discs reported that intradiscal pressure (IDP) gradually reduces with disc degeneration [17]. A compressive test on the nucleus demonstrated that the effective aggregate modulus decreases with increasing degeneration, whereas the permeability gradually increases [6].

Although numerous *in vivo* and *in vitro* experiments on disc degeneration have been conducted, the detailed distribution of the internal biomechanical parameters could not be measured by *in vivo* study and the different complex loading conditions for one sample could not be conducted through *in vitro* study because inevitable damage to the sample could not be prevented [12, 18]. With the rapid development of computer technology, researchers have adopted the finite element analysis (FEA) to simulate the biomechanical responses of lumbar spine. Compared with the elastic material model that does not have the capability to express fluid behavior, the poroelastic material model is more fit for describing the material property of spine components because fluid flow plays a key role in daily spinal physiological activities. Furthermore, accurate prediction of how the spine responds under complex loads has been proven using the lumbar spine modeled by poroelastic material [11, 18–21]. Changes in the geometrical and material parameters that occurred in different degenerated discs are investigated, and the results showed that the axial displacement, facet force, and total fluid loss are reduced with increasing degeneration [5, 22]. Results of the analysis of four intervertebral discs with different degenerated grades showed that the IDP is highest in flexion, and that IDP increases with the severity of degeneration [13]. The investigation of the processes that result in mechanical damage for different degenerated discs demonstrated that the number of cycles to failure sharply decreases with increasing degeneration and that the damages in healthy and moderately degenerated discs initiate at the posterior inner annulus and propagate outwards toward its periphery, whereas in serious degenerated case, damage initiates at the posterior outer annulus and propagates circumferentially [23].

Most finite element (FE) studies in the current literature have simulated diurnal working or constant load. The distribution of internal stress, changes in displacement, and number of repeated lifting that can lead to disc damage under diurnal working or constant load have been investigated in detail [24–26]. However, knowledge of the responses of lumbar spine when the working load consists of different resting modes is minimal. Such investigation can help people identify how and when to have a rest and how long each rest takes will be favorable for restoring degenerated intervertebral disc.

Therefore, this study aimed to explore appropriate working-resting modes in diurnal activity for healthy people and for the patients with degenerated intervertebral discs by creating four poroelastic FE models of lumbar spinal segments L2-L3 and then investigating their biomechanical responses under different loading conditions. Furthermore, establishing a FE model that can precisely express mechanical properties is important. Accurate anatomic structural data are required in constructing such a FE model. To meet the requirement, four sets of CT images of typical lumbar spines with different grades of disc degeneration were selected. This study may provide ideal working-resting modes for different grades of disc degeneration and serve as the theoretical basis for prevention and treatment of disc degeneration.

## Methods

### Specimens of lumbar spines with different grades of degenerated intervertebral discs

The study was approved by the ethics committee of the First Hospital of Jilin University. CT data from different individuals may have differences in terms of size, or other differences due to physiological variabilities. Thus, four volunteers with typical grades of different degenerated intervertebral discs were selected based on the procedure of Wilke *et al.* [9], which classifies different degenerative conditions into four grades. Written informed consent for participation in the study was obtained from the participants. Based on the CT images, the degrees of disc degeneration of the above four lumbar spines were sorted into Grades 0 to 3, based on the height loss as well as the number and length of osteophytes.

**Fig. 1:**
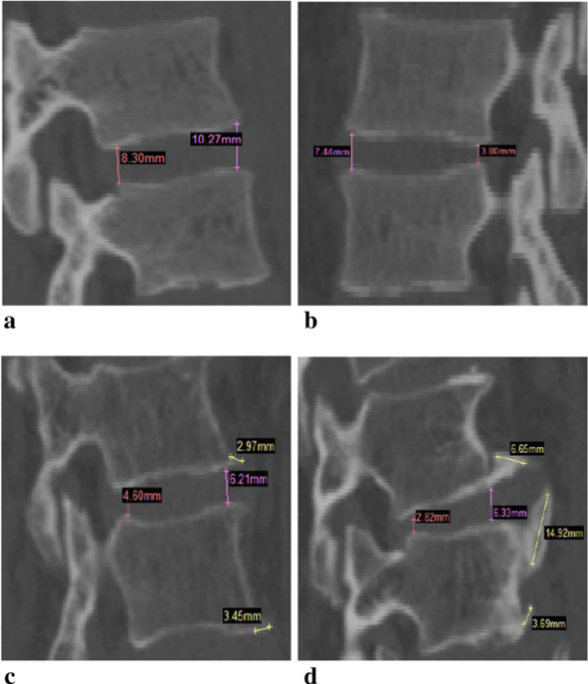
The sagittal section images of L2-L3 spinal segment with different grades of disc degeneration. **a** Grade 0: healthy disc with the leading edge height of lumbar 10.27 mm and the trailing edge height 8.30 mm, **b** Grade 1: mildly degenerated disc with the leading edge height of lumbar 7.44 mm and the trailing edge height 3.80 mm, **c** Grade 2: moderately degenerated disc with the leading edge height of lumbar 6.21 mm and the trailing edge height 4.60 mm, the osteophytes were between 2.97 mm and 3.45 mm, **d** Grade 3: seriously degenerated disc with the leading edge height of lumbar 6.33 mm and the trailing edge height 2.82 mm, and the maximum osteophyte was 14.92 mm

Figure 1 showed the following: (a) a 21-year-old healthy volunteer with normal disc height and good sclerotin, (b) a 23-year-old youngster with mildly decreased height, (c) a 47-year-old with moderately decreased disc height and mild osteophytes, (d) a 65-year-old man who suffered from serious osteophyte and disc height reduction.

### Establishment of FE models

**Fig. 2:**
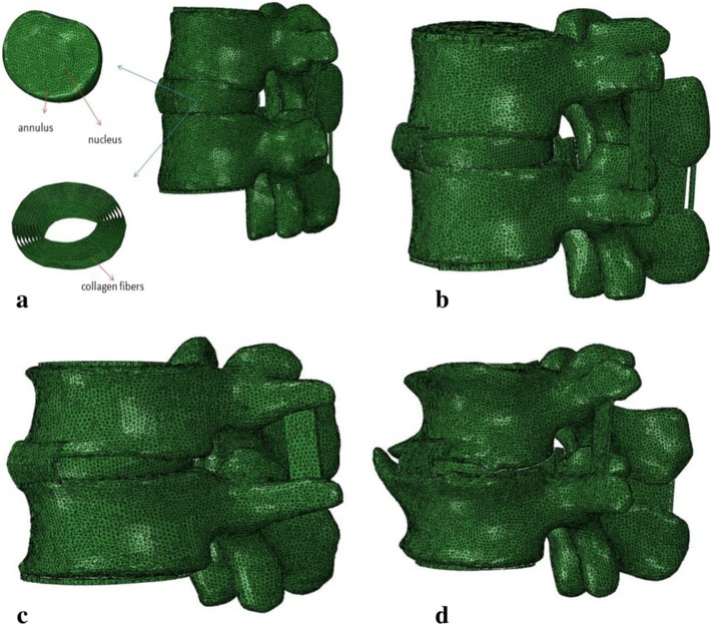
FE models of four different grades of degenerated discs. **a** Grade 0: healthy model with detailed view of the intervertebral disc included the collagen fibers which were embedded in the annulus ground substance in radial direction around the nucleus in eight layers, **b** Grade 1: mildly degenerated disc model, **c** Grade 2: moderately degenerated disc model, **d** Grade 3: seriously degenerated disc model

Four poroelastic FE models of the functional spinal units L2-L3 with different grades of degenerated intervertebral discs were created with MIMICS 10.01 (Materialise, Leuven, Belgium) and ABAQUS 6.11 (Simulia, Providence, USA). Most of the geometrical details of the models were acquired from CT scanning with a slice thickness of 0.7 mm. Spinal structures that were not displayed by CT imaging, such as ligaments, were reestablished through anatomical locations and actual shapes. Entitative ligament models were created and assembled between the vertebral bodies by boolean operation in MIMICS. The annulus fibrosus was modeled as a fiber-reinforced composite. Fibers were embedded in the annulus matrix in concentric rings around the nucleus in eight layers, which were endowed with membrane elements with rebar layers [10, 27]. The angulations of the crisscross fiber layers varied from ±24°to the horizontal plane ventrally to ±46°at the dorsal side [28, 29]. Surface structures were imported into ABAQUS to convert the surface mesh into volumetric mesh and subsequently for solving. The FE models (*i.e.* Grades 0 to 3) were shown in Fig. 2.

### Material properties

All structures were endowed with biphasic materials such as solid and saturated fluid phases, except collagen fibers, ligaments, and facets (Table 1). The annulus ground substance was assumed to have no change during degeneration [13, 30, 31]. Previous experiments and observations suggested that the nucleus material became similar to the annulus matrix owing to disc degeneration, which enhanced the stiffness of the nucleus, increased permeability and decreased water content [6, 11]. Thus, the nucleus material in Grade 3 was set to be identical with the annulus ground substance. Nucleus materials in Grades 1 and 2 were both obtained from linear interpolation based on Grades 0 and 3 [6, 11, 13]. The solid phase of annulus ground substance and nucleus were represented as Neo-Hookean hyperelastic material and parameters were chosen based on literature [20, 21, 32]. The stiffness of the fiber proportionally decreased from outside to inside for every two fiber layers and the proportion varied from 1 to 0.65 [10]. The nonlinear behaviors of the ligaments and facets were modeled with incompressible, hyperelastic Neo-Hookean formulation. Material parameters were obtained from the stress–strain curves in the previous experimental study [33].

**Table 1:** Mechanical properties of poroelastic materials in the FE models for all L2-L3 segments

a. Elastic properties of the lumbar except nucleus pulposus				
Model	Material model	Material property		
		Elastic modulus (MPa)	Poisson^’^s ratio (μ)	
Cancellous bone	Linear-elastic	100	0.2	
Cortical bone	Linear-elastic	10000	0.3	
Cartilage endplates	Linear-elastic	5	0.1	
Annular fiber	Linear-elastic	357-550	0.3	
Annulus ground substance	Hyper-elastic	Neo-Hookean C_10_ = 0.315, D = 0.688		
Ligaments/Facet	Hyper-elastic	Fitting from test data		
The linear-elastic properties of cancellous bone, cortical bone, and cartilage endplates were based on Schmidt *et al.* [19]. The linear-elastic properties of annular fiber were based on Xu *et al.* [10]. The hyper-elastic properties of annulus ground substance were based on Schmidt *et al.* [20], Schmidt *et al.* [21], and Galbusera *et al.* [32]. The hyper-elastic properties of ligaments/facet were based on Sharma *et al.* [33].				
b. Porous properties of the lumbar spine except nucleus pulposus				
Material	Permeability (m^4^/Ns)	e_0_	M (Eq. (1))	
Cancellous bone	k = 1e^−13^	0.4		
Cortical bone	k = 1e^−20^	0.02		
Cartilage endplates	k_0_ = 7e^−15^	4	10	
Annulus ground substance	k_0_ = 3e^−16^	2.33	12	
The porous properties of cancellous bone, cortical bone, cartilage endplates, and annulus ground substance were based on Argoubi and Shirazi-Adl [34].				
c. Poroelastic material properties of all the nucleus pulposus				
Nucleus pulposus	Hyper-elastic	Poroelastic		
	Neo-Hookean	Permeability (m^4^/Ns)	e_0_	M (Eq. (1))
Grade 0	C_10_ = 0.12, D = 2.475	k_0_ = 3e^−16^	4	10
Grade 1	C_10_ = 0.185, D = 1.88	k_0_ = 5e^−16^	3.44	10
Grade 2	C_10_ = 0.25, D = 1.285	k_0_ = 7e^−16^	2.88	10
Grade 3	C_10_ = 0.315, D = 0.688	k_0_ = 9e^−16^	2.33	10
The hyper-elastic properties of nucleus pulposus for Grade 0 to 3 were based on Schmidt *et al.* [13], Schmidt *et al.* [20], and Schmidt *et al.* [21]. The porous properties of nucleus pulposus for Grade 0 to 3 were based on Johannessen *et al.* [6], Massey *et al.* [11], and Argoubi and Shirazi-Adl [34].				

Permeability was set to be changed with void ratio and could be implemented directly in the Property Module of ABAQUS. The strain-dependent permeability formulation was taken from Argoubi and Shirazi-Adl [34]. The permeability *k* is dependent on void ratio *e*:

1 $$ k={k}_0{\left[\frac{e\left(1+{e}_0\right)}{e_0\left(1+e\right)}\right]}^2 \exp \left[M\left(\frac{1+e}{1+{e}_0}-1\right)\right] $$

where *k*_0_ is the initial permeability, *e*_0_ is the initial void ratio, and *M* is the constant used to match experimental measurements.

### Boundary and loading conditions

The six degrees of freedom of the nodes at the bottom of the inferior endplate of L3 were all constrained. The phenomenon of the healthy disc swelling because of osmotic potential was simulated by a 0.25 MPa boundary pore pressure at the outer surface of the spine [3, 5, 19]. Boundary pore pressure was linearly reduced to 0.1 MPa from the non-degenerated intervertebral disc to the seriously degenerated one because of consideration to the loss of proteoglycans caused by disc degeneration [22]. The Interaction Property “TIE” in ABAQUS was used to define all the surface to surface contacts in the FE models [35].

**Fig. 3:**
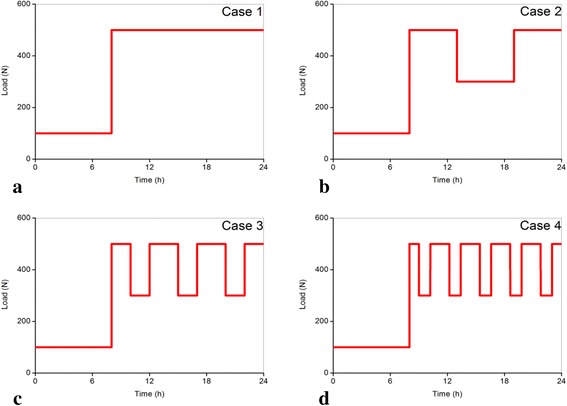
Time history of the applied compressive forces. **a** Load Case 1 with no rest in diurnal activity, **b** Load Case 2 with one-time rest in diurnal activity, **c** Load Case 3 with three-time rests in diurnal activity, **d** Load Case 4 with five-time rests in diurnal activity

**Fig. 4:**
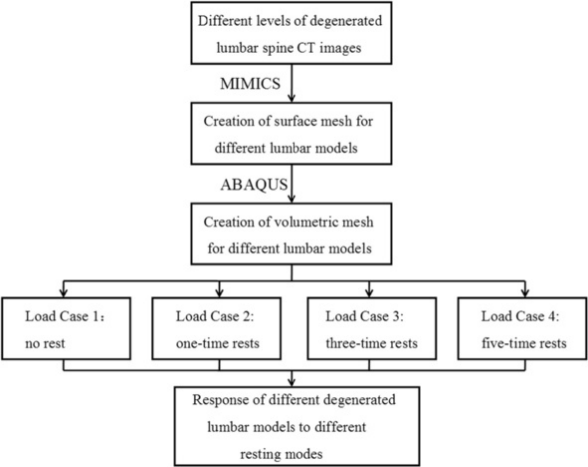
Schematic diagram of simulation process

The load was applied to a reference point above the upper vertebral body. This reference point was coupled to the superior endplate of L2 and all the degrees of freedom except axial translation were constrained. The loading amplitudes were classified into three types: 500 N to simulate the axial compressive force spine sustained during diurnal work as reference [20] reported; 300 N to represent the load when having a cozy rest such as sitting during daytime as reference [36] showed; and the night load of 100 N was taken as average axial compressive load on lumbar in supine position as reported by [20, 37]. Total sleeping time, working time, and resting time were assumed to be invariable at 8, 10, and 6 h, respectively. However, the resting frequency and each resting time during the total 6 h were not the same. Four loading conditions that represented four different resting modes were considered in this study. These conditions consisted of one-time long rest of 6 h in the noon; three-time moderate rests, in which each rest endured 2 h in the morning, noon and afternoon, respectively; and five-time short rests that each lasted 1.2 h and were averagely distributed in daytime. A mode that had no rest during daytime was employed for comparison. The four different loading conditions were illustrated in Fig. 3. The whole simulation process of lumbar spines with different levels of degenerated intervertebral discs was shown in Fig. 4.

### Model validation

Creep is an important characteristic in lumbar mechanical activity. Thus, comparing the changing creep parameters with the experiments can validate the accuracy of our FE model. To enhance the comprehensiveness and accuracy of the validation, two verifications of numerical analyses were conducted for two parts: one for validating the creep characteristic of intervertebral disc, in which the lumbar specimen was subjected to 1 MPa pressure for 20 min for comparison with the experiment [38], and the other for validating the strains of the vertebral bodies that changed with time under compressive force of 1000 N for 0.5 h for comparison with the *in vitro* study [39]. Additional details on the model validation and mesh sensitivity analyses were described in the Discussion section.

## Results

### Axial effective stress in the nucleus

Axial effective stress is the pressure sustained by the solid phase in the poroelastic model. The axial direction was the same as the loading direction.

The increased/decreased changing rates in this study were described by: increased/decreased changing rate = (Result of Case 2/3/4 - Result of Case 1)/Result of Case 1 × 100 %.

**Fig. 5:**
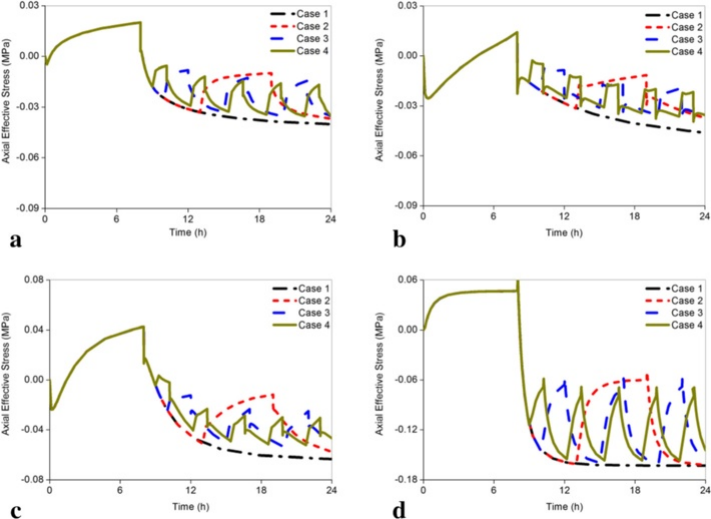
Comparison of the changes of the axial effective stress in the center of the nucleus subjected to different load cases for all models. Axial effective stress is the axial pressure sustained by the solid phase in the poroelastic model. Significant relationships were found between axial effective stress and intervertebral disc degeneration. The mechanical properties of disc could be better restored by the less axial effective stress in the center of the nucleus. **a** Grade 0, **b** Grade 1, **c** Grade 2, **d** Grade 3

Figure 5a-d showed the change of axial effective stress in the nucleus across all models. Results indicated that compared with Case 1 (no rest during daily activity), the axial effective stress in the nucleus of healthy disc decreased by 8.302 % in Case 2, 12.491 % in Case 3, and 12.939 % in Case 4 (Fig. 5a). Compared to the healthy disc, the mildly and moderately degenerated discs generated higher reductions in axial effective stress, while they decreased by 19.318 % and 8.959 % in Case 2, 22.075 % and 20.608 % in Case 3, 23.030 % and 25.831 % in Case 4 (Fig. 5b-c). For the seriously degenerated disc, the decreasing axial effective stress in Case 2 (0.565 %) was not obvious compared with reductions of 4.472 % in Case 3 and 11.242 % in Case 4 (Fig. 5d). Following the severity of degeneration, the axial effective stress in the nucleus increased across all cases.

### Axial effective stress in the posterior annulus

**Fig. 6:**
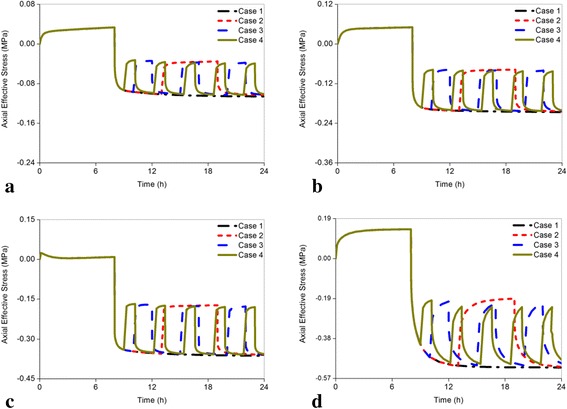
Comparison of the changes of the axial effective stress in the posterior annulus subjected to different load cases for all models. Compared with the changing rates of axial effective stress in the center of the nucleus under Cases 2 to 4, the changing rates of axial effective stress in the posterior annulus under Cases 2 to 4 were low. **a** Grade 0, **b** Grade 1, **c** Grade 2, **d** Grade 3

Figure 6a-d exhibited the axial effective stress changes in the posterior annulus across all models. The least axial effective stress in the posterior annulus occurred in Case 4 for all models. However, compared with axial effective stress in the nucleus, the degrees of reduction from Cases 2 to 4 were not obvious. The highest reduction was found in the seriously degenerated disc, in which the axial effective stress ranged from 0.517 MPa in Case 1 to 0.481 MPa in Case 4. However, in the healthy, mildly, and moderately degenerated discs the axial effective stress was only reduced by 4.289, 2.462, and 1.741 % in Case 4. Among the cases, the axial effective stress in the posterior annulus increased gradually with the escalation of disc degeneration.

### Pore pressure in the nucleus

**Fig. 7:**
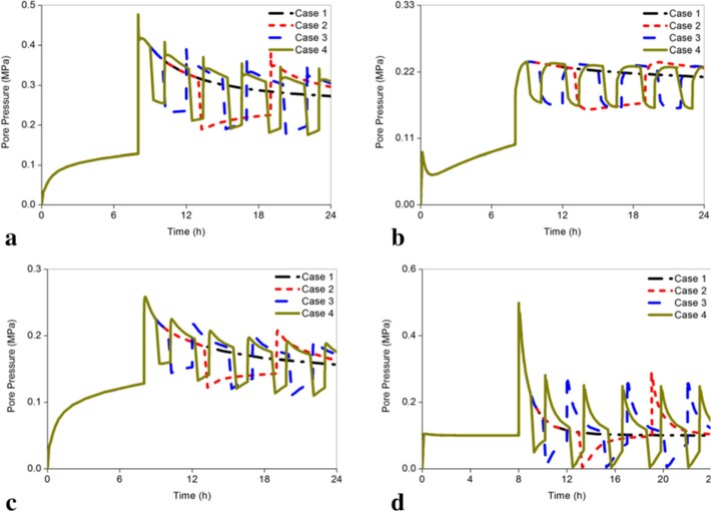
Comparison of the changes of the pore pressure in the center of the nucleus subjected to different load cases for all models. Pore pressure works to resist mechanical stress in the solid matrix of disc by hydration of molecules. Proper increasing pore pressure can reduce the load sustained by the solid phase, which is beneficial to restoration of degenerated disc. **a** Grade 0, **b** Grade 1, **c** Grade 2, **d** Grade 3

Figure 7a-d indicated the pore pressure changes in the center of the nucleus. The highest pore pressure for the healthy disc was found in Case 4 (0.309 MPa), followed by Case 3 (0.305 MPa), Case 2 (0.296 MPa), and Case 1 (0.273 MPa) (Fig. 7a). Except for in Case 2, which increased by 7.072 %, no significant differences among the other cases for the mildly degenerated disc were derived (Fig. 7b). In contrast to the two less degenerated discs, the pore pressure increased in Case 3 (9.592 %) and Case 4 (11.829 %) for the moderately degenerated disc (Fig. 7c). For the seriously degenerated disc, the pore pressure increased by 28.001 % in Case 4, but only increased by 2.005 % in Case 2 (Fig. 7d). When degeneration increased, the pore pressure across all cases decreased gradually because of the loss of proteoglycans.

### Radial displacement

**Fig. 8:**
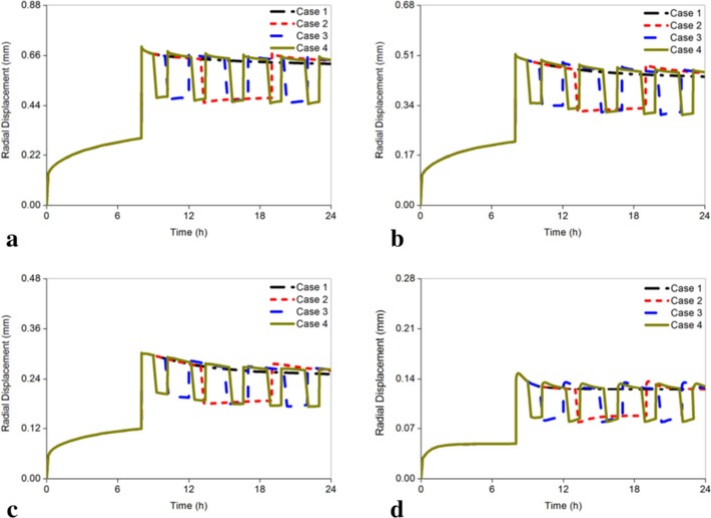
Comparison of the changes of the radical displacement subjected to different load cases for all models. The radial displacement is closely related to disc degeneration. The maximum changing magnitude in radial displacement was not obvious in comparison with the changing magnitudes in axial effective stress and pore pressure. **a** Grade 0, **b** Grade 1, **c** Grade 2, **d** Grade 3

Figure 8a-d showed the change of radial displacement for all models (the radial direction was perpendicular to coronal plane). The healthy disc showed that the range of radial displacement was 0.624 mm-0.642 mm from Cases 1 to 4 (Fig. 8a). Even with the increasing trend, the values in all the four cases were almost the same at the end of the day, of which the differences for the cases were smaller than 0.015 mm. Similar to the healthy disc, the maximum differences under different cases for the remaining degenerated discs were 0.015 mm, 0.011 mm, and 0.005 mm in the mildly, moderately, and seriously degenerated model, respectively (Fig. 8b-d). Furthermore, based on the severity of degeneration, the radial displacement decreased in all cases.

### Fluid loss

**Fig. 9:**
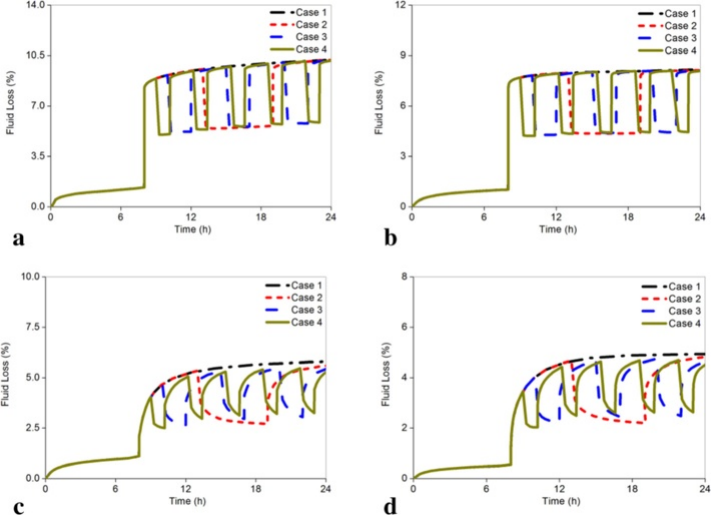
Comparison of the changes of the disc fluid loss subjected to different load cases for all models. The fluid loss could reflect the capacity of convective transport of solutes within the intervertebral disc, and low fluid loss is closely related to disc degeneration. **a** Grade 0, **b** Grade 1, **c** Grade 2, **d** Grade 3

Figure 9a-d showed the changes of the average disc fluid loss between annulus and nucleus for all models. The results indicated that fluid loss increased when bearing loads and decreased with degeneration due to the inability of the disc to imbibe the lost fluid [11]. For the healthy disc, the maximum average fluid loss was 10.214 % in Case 1, followed by 10.194 % in Case 2, 10.184 % in Case 3, and 10.180 % in Case 4. These cases were accompanied with a declining trend from Cases 2 to 4 (Fig. 9a). The maximum decrease in the changing rate of fluid loss for overall degenerated discs was 9.122 % in the moderately degenerated disc in Case 4, followed by 8.446 % in the seriously degenerated disc in Case 4 (Fig. 9c-d). Nevertheless, the maximum changing magnitude was not obvious in comparison with the axial effective stress and pore pressure.

### Sensitivity analyses of material parameters in FEA

The sensitivity of the results to the values assigned to the parameters of the Grade 0 model was analyzed as well, such as changes in the permeability and boundary pore pressure. The results showed that when permeability was doubled, the pore pressure in the nucleus increased by 7.531 % in Case 2, 9.494 % in Case 3 and 11.332 % in Case 4. Furthermore, when the boundary pore pressure was reduced to 0.2 MPa, the pore pressure in the nucleus increased by 10.281 % in Case 2, 12.383 % in Case 3, and 14.521 % in Case 4. Therefore, the changing trends were similar and the conclusions obtained were the same as those of our study with normal material values. This indicated that moderately changing parameter values had slight effects on the conclusions.

## Discussion

The effects of different working-resting modes on different levels of degenerated intervertebral discs were investigated in this study. The cases designed in current study could represent the actual working-resting conditions in different professions. Case 1 was served as a control; Case 2 represented the normal working-resting condition that works in the morning and afternoon sessions and rests at noon; Case 3 represented the professions, such as teachers who enjoy a rest period after several classes; and Case 4 represented the professions whose working and resting times are not fixed, such as the group of students or waiters who have short rest periods after their respective short work periods. Meanwhile, 10 h working time, 6 h resting time, and 8 h sleeping time in one day conform to normal people’s living habit. Therefore, all these designed cases were in accordance with most of real-life cases. Thus, this study could provide a basis for clinical studies on different professions.

The predicted radial displacement results of the non-degenerated segments in Case 1 agreed well with the *in vitro* measured radial displacement. The average deformation of disc in the anterior region is 0.86 mm with the range of 0.46 mm-1.34 mm under 500 N [40]. Our predicted radial displacement of 0.624 mm (Fig. 8a) was within this range and near the mean value. Another numerical analysis showed that the peak and ending pressure of the normal disc could reach 0.47 MPa and 0.26 MPa after 16 h of calculation under constant 500 N [20], which were similar to our results in Case 1 (Fig. 7a). The axial effective stresses in mildly, moderately and seriously degenerated discs were reported to be in the ranges of 0.10 ± 0.10 MPa, 0.14 ± 0.24 MPa, and 0.25 ± 0.29 MPa respectively after a 16 h diurnal activity [5]. Our predicted results in Case 1 under the same condition were all within this ranges, which was 0.045 MPa in mildly degenerated model, 0.065 MPa in moderately degenerated model, and 0.161 MPa in seriously degenerated model, respectively (Fig. 5b-d). These comprehensive comparisons with previous experiments and FEA in terms of displacement, pore pressure, and axial effective stress validated the accuracy of our results.

**Fig. 10:**
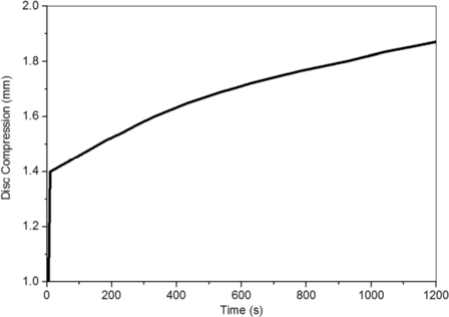
Temporal change of disc compression displacement in FE model of Grade 0 under 1 MPa pressure

**Fig. 11:**
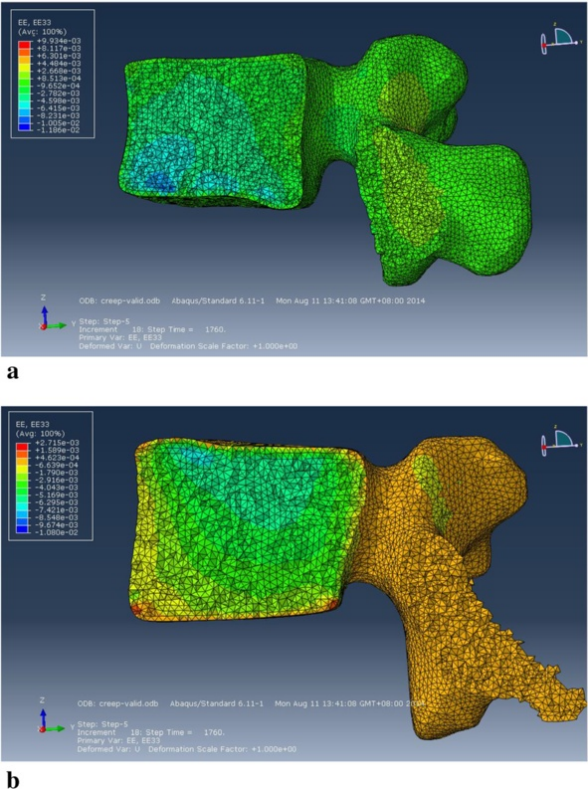
Contour plot of strain on the vertebral body during compressive force of 1000 N. **a** L2, **b** L3

More details regarding the validation and mesh sensitivity analyses of our FE models were discussed according to compare with *in vitro* experiments. Validation 1: the response of disc compression was compared with the experimental measurements under the same boundary condition [38]. The elastic deformation of the disc measured by the experiment was 1.35 mm, then the creep deformation increased to 1.85 mm. The deformations in the disc were 1.4 mm and 1.88 mm in our FEA (Fig. 10). These results proved that the predicted displacements were in satisfactory agreement with the measurements. Validation 2: the elastic and creep strains predicted by our FE model were compared with those of the *in vitro* experiment under a compressive force of 1000 N [39]. In the 0.5 h in the vitro experiment, creep deformations in the anterior, middle, and posterior vertebral cortex averaged 4331, 1629 and 614 micro-strains and elastic deformations in the anterior, middle and posterior vertebral cortex averaged 6936, 3014, 3879 micro-strains, respectively. The experimental results showed larger average deformations in the anterior vertebral body compared with the posterior part. This observation was the same as our predicted results (Fig. 11). Our predicted strains in the anterior, middle, and posterior parts of L2 and L3 (Table 2) were very close to the mean values that were tested by the *in vitro* study. Mesh sensitivity analyses: the sensitivity analyses of the mesh size were also done using the IDP results generated by 7.5 Nm flexion moment, which simulated the experiment [41]. Every model was remeshed to establish five different models with different mesh densities (Table 3). The IDP increased with the reduction in the mesh density for nearly all models. For Grades 0 to 2, model C generated slight differences to models A and B. However, the number of elements as well as the computational time was reduced effectively and the result of model C of Grade 0 was closer to that of the experiment [41]. The results of models D and E were slightly unstable. Thus, model C was chosen for the healthy, mildly and moderately degenerated discs. For Grade 3, model C was highly different to model A compared with model B, and Models D and E also produced unstable results; thus, model B was chosen. These showed that the choices of mesh sizes, which were based on relatively low computational expense but higher computational precision, were reasonable. All of the abovementioned model validations and mesh sensitivity analyses illustrated that our FE models were well validated and can be used to investigate the effects of different working-resting modes on different degenerated intervertebral discs.

**Table 2:** The predicted elastic and creep strains in different regions of this FE model under compressive force of 1000 N for 0.5 h

	Anterior	Middle	Posterior
a. The predicted elastic and creep strains in different regions of L2			
Elastic strain	6899	3006	3772
Creep strain	4964	2555	486
b. The predicted elastic and creep strains in different regions of L3			
Elastic strain	6706	2935	3642
Creep strain	4064	2325	473

(Units are micro-strains, where 10000 micro-strains = 1 % strains.)

**Table 3:** Comparisons of element size, number, and IDP of our different levels of degenerated models with different mesh densities

		The edge length of elements (mm)	Number of elements	IDP (MPa)
Grade 0	Model A	0.5-0.7	3903319	0.262
	Model B	0.7-0.9	1659902	0.264
	**Model C**	**0.9-1.1**	**1216302**	**0.266**
	Model D	1.1-1.3	999088	0.289
	Model E	1.3-1.5	737770	0.637
Grade 1	Model A	0.5-0.7	3324073	0.224
	Model B	0.7-0.9	1325175	0.227
	**Model C**	**0.9-1.1**	**661800**	**0.229**
	Model D	1.1-1.3	373308	0.234
	Model E	1.3-1.5	243281	0.235
Grade 2	Model A	0.5-0.7	3256612	0.151
	Model B	0.7-0.9	1236574	0.166
	**Model C**	**0.9-1.1**	**629499**	**0.162**
	Model D	1.1-1.3	369033	0.176
	Model E	1.3-1.5	237352	0.15
Grade 3	Model A	0.5-0.7	3066937	0.107
	**Model B**	**0.7-0.9**	**1233808**	**0.109**
	Model C	0.9-1.1	599615	0.114
	Model D	1.1-1.3	350707	0.103
	Model E	1.3-1.5	232444	0.119

(The models marked in bold were those used in this study.)

**Fig. 12:**
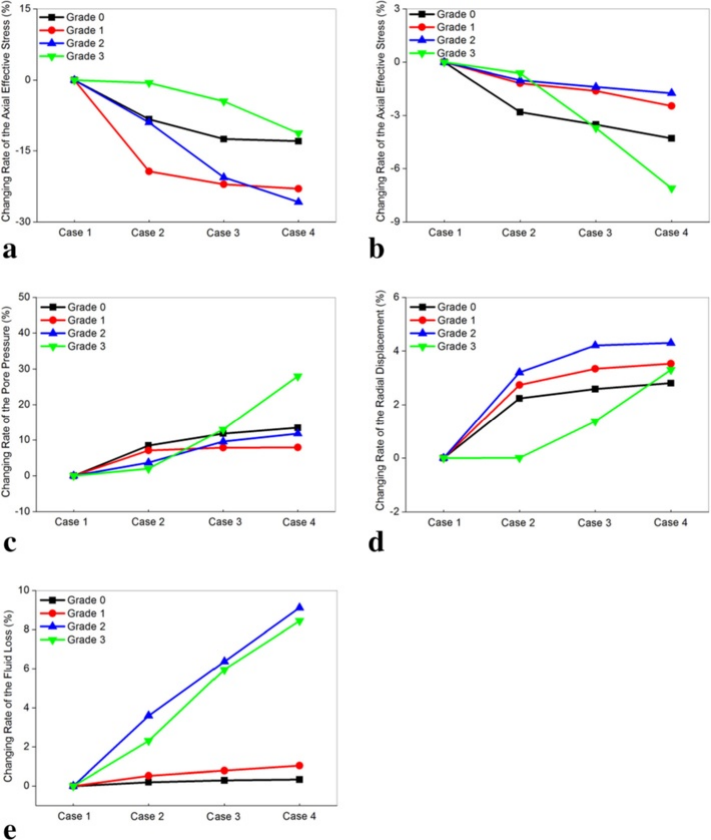
The changing rate under different resting frequency. The differences in the changing rate of axial effective stress and pore pressure under different resting frequency were significant, and the rest parameters did not exhibit obvious differences. Because the coordinate axis scale of radial displacement was small, a large difference in radial displacement between Grade 2 and 3 was displayed; actually, both of the maximum changing rates were less than 5 % from case 1 to 4 for these two models. **a** The axial effective stress in the center of nucleus, **b** The axial effective stress in the posterior annulus region, **c** The pore pressure in the center of nucleus, **d** The radial displacement, **e** The fluid loss

With increasing resting frequency, the axial effective stress in the nucleus and posterior annulus and disc fluid loss decreased (Figs. 5a-d, 6a-d, 9a-d), whereas the pore pressure in the center of the nucleus and radial displacement increased gradually (Figs. 7a-d and 8a-d). Meanwhile, all the changing rates of biomechanical parameters varied under different resting frequencies (Fig. 12a-e). As Fig. 12a shown, the results of the healthy and mildly degenerated discs indicated that both of the highest changing rates of the axial effective stress in the nucleus occurred in Case 2, followed by Case 3 and Case 4, and the differences between the changing rates in Case 3 and Case 4 were few. Nevertheless, in the moderately degenerated disc, the highest changing rate happened in Case 3. The seriously degenerated disc appeared completely different from the less degenerated discs, in which the axial effective stress in Case 2 almost had no difference with Case 1 and the highest changing rate occurred in Case 4. Thus, the changing rate of axial effective stress for the seriously degenerated disc could be considered to increase with the increase in resting frequency. In conclusion, one-time rest may have slight effects on the reduction of the axial effective stress in the nucleus; however, five-time rests may be the best choice for the seriously degenerated disc according to this analysis. From Fig. 12b and Fig. 6a-d, all changing percentages of the axial effective stress in the posterior annulus changed slightly from Cases 2 to 4 except for the seriously degenerated disc. Axial effective stress is the pressure sustained by the solid phase in the poroelastic structure. The highest axial effective stress was predicted to occur in the most serious degeneration. Axial effective stress may result in a lumbar disc herniation that is generally found in young patients, delamination and tear have higher incidence rates among the elderly [5, 42, 43]. Therefore, the analysis demonstrated that in a fixed period of resting time, rest with higher frequency could be much more favorable for reducing the axial effective stress.

The changing rate of pore pressure in the center of the nucleus was reduced with increasing frequency for the healthy, mildly degenerated discs (Fig. 12c). For the moderately degenerated disc, the highest changing rate occurred in Case 3 as the axial effective stress. On the contrary, the changing rate of pore pressure for the seriously degenerated disc increased when the resting frequency was on the rise, of which the increasing percentage of Case 2 was nearly 2 %, but in Case 4 it reached 28.001 %. Pore pressure resists mechanical stress in the solid matrix of the disc by hydration of molecules and the proteoglycans will be extruded during high pressure. This could explain the phenomenon where a suddenly compressive force leads to maximum pore pressure at 8 h and then dissipate gradually. Thus, proper increasing pore pressure can decrease the load sustained by the solid phase, which was beneficial to restoration of the degenerated disc [11]. Therefore, the results of pore pressure advised patients to have more resting frequency under fixed resting time such as in Case 4. Subsequently, as shown in Fig. 12d, the radial displacement for all discs expressed an adverse trend that negatively affected the restoration of the degenerated disc when the resting frequency was on the rise. However, the maximum changing rate was less than 5 %. The disc fluid loss also indicated very slight differences among the different resting frequencies for healthy and mildly degenerated discs and a slight distinction in the seriously degenerated disc with less than 10 % as shown in Fig. 12e. The changing rates of radial displacement and fluid loss in different cases were minimal; thus, the restoration of the degenerated disc may not be influenced too much by different resting modes.

Results from the abovementioned phenomena demonstrated that for the normal and mildly degenerated discs, all the changing rates of biomechanical parameters reduced gradually with the increase in the resting frequency. However, the highest changing rate for moderately degenerated disc occurred in Case 3. Unlike the less degenerated discs, all the changing rates in the seriously degenerated disc started to increase from Cases 2 to 4; thus, almost no help was available in Case 2 to restore the degenerated disc. Healthy people and patients with degenerated discs were advised to divide fixed resting time into short and separated periods as much as possible, as illustrated by Case 4. In this way, axial effective stress could be reduced and pore pressure could be increased to a maximum, which were beneficial to restoration of the damaged disc. Additionally, relatively less resting frequency, such as in Case 3, also enhanced the efficiency of the restoration of healthy, mildly degenerated, and especially for moderately degenerated discs under the circumstance that too many resting frequencies were unavailable. For the serious patient, a slight effect of one-time rest was noted even though the resting time was sufficiently long enough.

Previous *in vitro* studies suggested that the intervertebral disc is a tissue with small cell proliferation and regeneration capacity both in annulus fibrosus and nucleus pulposus [44–46]. Cell proliferation and regeneration capacity can be increased by external stimulation to promote the restoration of intervertebral discs. Thus, more biological repair mechanisms can be taken into account to relieve the pain caused by disc degeneration. Running exercise can serve as a better external stimulation to increase extracellular matrix production and cell number in the annulus fibrosus [47, 48]. Different working-resting modes that were beneficial to different levels of degenerated intervertebral discs have been predicted in current study. Thus, combining with the phenomenon where moderate exercise has positive effects on cell proliferation in intervertebral disc, exercise-resting modes that are beneficial to different levels of degenerated intervertebral discs could also be determined by the similar numerical simulations.

However, excessive running does not always enhance proliferation, and that the decrease in progenitor proliferation seen in long-term running is possibly mediated by mechanisms involving a stress response in the animal [49]. Cell proliferation could be promoted only under moderate exercise; excessive exercise may lead to negative effects [50]. Thus, determining different exercise-resting modes that are beneficial to different degenerated intervertebral discs is crucial. Although different working-resting modes that were fit for different degenerated models have been predicted in the current study, the external load sustained by the lumbar spine in working and exercising conditions differed. Hence, in our future work, a number of new running-resting cases will be designed to investigate their positive effects on the different levels of degenerated intervertebral discs.

Numerous assumptions in the creation of our FE models persisted. Focal clefts and cavities were neglected in modeling degenerated discs. Meanwhile, the interaction condition “TIE” was used to link the facet joint and vertebral body because of the convergence problem, which was slightly unrealistic. Finally, the muscular constitution was not simulated in the current models. The muscular tissue may not only bear parts of external loads, but may also influence the restoration of intervertebral disc. Thus, a lack of muscular constitution in the FE models can exert certain effects on the predicted results. However, the conclusions were obtained by comparing the mechanical responses of different degenerated models, and this study emphasized observation of the effects of different working-resting modes on different levels of degenerated models. The effects of muscular constitution were not considered for all different degenerated models, and exterior geometry and interior material changes caused by degeneration were the main concern for all the different degenerated models. Therefore, the conclusions may not significantly influenced by the exclusion of the muscular constitution. Although our FE models were based on the abovementioned assumptions, the model validation ensured the accuracy of our models. Thus, the accuracy of our results can be vouched. Moreover, meaningful clinical advice can be obtained because of the precise modeling and analytical method implemented.

## Conclusions

In summary, the biomechanical responses of different degenerated intervertebral discs under different working-resting modes were analyzed using three-dimensional poroelastic FE models. The predicted responses revealed that to keep the intervertebral disc healthy, the fixed resting time should be divided into separated periods as much as possible for all intervertebral discs with different grades of degeneration, especially for the seriously degenerated intervertebral disc because one long resting period showed minimal effect on the aim of decreasing the harmful biomechanical responses to the lumbar.

## Abbreviations

IDP: Intradiscal pressure
FEA: Finite element analysis
FE: Finite element
